# Covering the Last Kilometer: Using GIS to Scale-Up Voluntary Medical Male Circumcision Services in Iringa and Njombe Regions, Tanzania

**DOI:** 10.9745/GHSP-D-15-00151

**Published:** 2015-09-10

**Authors:** Hally Mahler, Sarah Searle, Marya Plotkin, Yusuph Kulindwa, Seth Greenberg, Erick Mlanga, Emmanuel Njeuhmeli, Gissenje Lija

**Affiliations:** ^a^​ Jhpiego, Dar Es Salaam, Tanzania; ^b^​ Jhpiego, Baltimore, MD, USA; ^c^​ United States Agency for International Development (USAID), Dar Es Salaam, Tanzania; ^d^​ USAID, Washington, DC, USA; ^e^​ Tanzania Ministry of Health and Social Welfare, Dar Es Salaam, Tanzania

## Abstract

**Background:**

Based on the established protective effect of voluntary medical male circumcision (VMMC) in reducing female-to-male HIV transmission, Tanzania's Ministry of Health and Social Welfare (MOHSW) embarked on the scale-up of VMMC services in 2009. The Maternal and Child Health Integrated Project (MCHIP) supported the MOHSW to roll out VMMC services in Iringa and Njombe, 2 regions of Tanzania with among the highest HIV and lowest circumcision prevalence. With ambitious targets of reaching 264,990 males aged 10–34 years with VMMC in 5 years, efficient and innovative program approaches were necessary.

**Program Description:**

Outreach campaigns, in which mobile teams set up temporary services in facilities or non-facility settings, are used to reach lesser-served areas with VMMC. In 2012, MCHIP began using geographic information systems (GIS) to strategically plan the location of outreach campaigns. MCHIP gathered geocoded data on variables such as roads, road conditions, catchment population, staffing, and infrastructure for every health facility in Iringa and Njombe. These data were uploaded to a central database and overlaid with various demographic and service delivery data in order to identify the VMMC needs of the 2 regions.

**Findings:**

MCHIP used the interactive digital maps as decision-making tools to extend mobile VMMC outreach to “the last kilometer.” As of September 2014, the MOHSW with MCHIP support provided VMMC to 267,917 men, 259,144 of whom were men were aged 10–34 years, an achievement of 98% of the target of eligible males in Iringa and Njombe. The project reached substantially more men through rural dispensaries and non-health care facilities each successive year after GIS was introduced in 2012, jumping from 48% of VMMCs performed in rural areas in fiscal year 2011 to 88% in fiscal year 2012 and to 93% by the end of the project in 2014.

**Conclusion:**

GIS was an effective tool for making strategic decisions about where to prioritize VMMC service delivery, particularly for mobile and outreach services. Donors may want to consider funding mapping initiatives that support numerous interventions across implementing partners to spread initial start-up costs.

## Abstract (precis)

Interactive GIS maps created by overlapping facility data including roads and infrastructure with population and service delivery data permitted strategic deployment of mobile voluntary medical male circumcision (VMMC) services to underserved rural communities. The percentage of VMMCs performed in rural areas jumped from 48% in 2011 to 93% in 2014.

## BACKGROUND

Voluntary medical male circumcision (VMMC) has been shown to reduce female-to-male HIV transmission by approximately 60% in randomized controlled trials.^1^^-^^3^ Cost and impact modeling has suggested that rapid scale-up of VMMC among men ages 15–49 years would drastically reduce HIV transmission.^4^

In 2009, the Ministry of Health and Social Welfare (MOHSW) of Tanzania incorporated VMMC into its national prevention strategy, targeting males aged 10–34 years, particularly in 11 regions where male circumcision was low. Although the national prevalence of male circumcision among 15- to 49-year-olds was 67% in 2009, it was as low as 23% in some regions of the country.^5^

**FIGURE 1:**
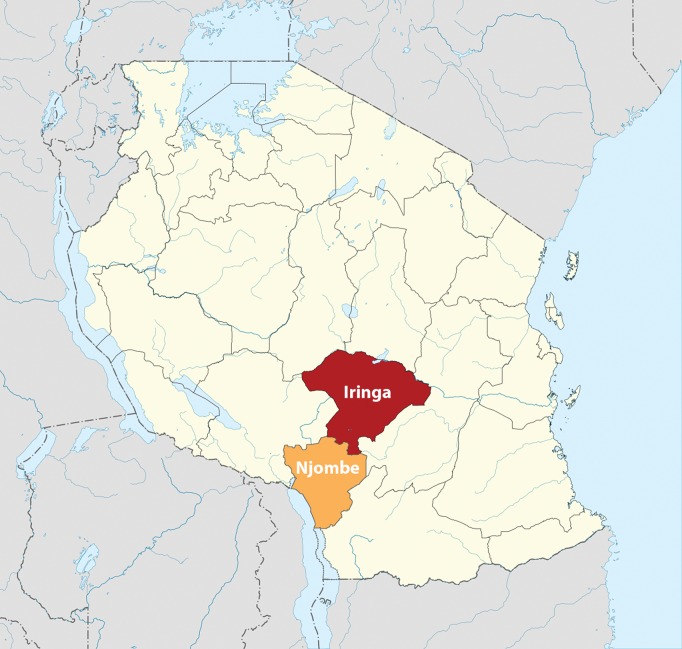
Voluntary Medical Male Circumcision Focus Regions in Tanzania Map image adapted from Sémhur/Wikimedia Commons/CC-BY-SA-3.0.

Tanzania’s “National Strategy for Male Circumcision for HIV Prevention (2010–2015)” set a target for 2.8 million boys and men aged 10–34 years to receive VMMC services in 11 of the country’s 34 regions. In Iringa and Njombe (which, at the time of project inception, were a single region called Iringa), the national strategy specified that 264,990 males aged 10–34 years should receive VMMC by 2015 (Figure 1).^6^ At the time, Iringa had one of the lowest male circumcision prevalence rates (29.1%) and the highest HIV prevalence (15.7%) of any region in the country.^5^^,^^6^

The Maternal and Child Health Integrated Project (MCHIP), led by Jhpiego and funded by the United States President’s Emergency Plan for AIDS Relief (PEPFAR) through the United States Agency for International Development (USAID), supported the MOHSW to roll out VMMC services in Iringa (and after partition in 2014, when the regions became separate, in Iringa and Njombe). MCHIP provided support in a number of areas, including training health care providers, providing commodities and equipment to health facilities, facilitating demand creation activities, guiding logistics, and providing technical and managerial support in quality assurance, supervision, and mentoring to the regional and district medical authorities. In addition, in the first 9 months of the program, MCHIP focused on assisting the regions to establish fixed VMMC sites where routinely scheduled services were offered several days a week.

The purpose of this article is to describe the program’s evolving use of geographic information systems (GIS) technology to strategically plan and implement outreach campaigns in order to extend VMMC services to remote rural areas and achieve rapid scale-up.

## PROGRAM DESCRIPTION

As in many other countries rolling out VMMC with PEPFAR support, the VMMC program in Tanzania set very ambitious targets. In the first year of implementation (October 2009 through September 2010), 22,970 males received VMMC services in Iringa and Njombe. While improvements in efficiency allowed the program to almost double coverage to 42,667 males in the following year, it was clear that service delivery models needed to be even more efficient if the project were to reach the regional target of 264,990 men by 2015.

Fixed sites (within public health facilities) offered VMMC services on scheduled days to clients willing to seek services at central locations, but the project needed to bring services out of urban areas and into rural areas where demand was high but access to VMMC was low. To respond to this need, VMMC service delivery models in Iringa and Njombe evolved to include campaigns and mobile sites over time (Box).

**BOX. Voluntary Medical Male Circumcision (VMMC) Service Delivery Definitions**

**Routine services:** Services provided on a regularly scheduled basis (may be at fixed sites or outreach sites).

**Campaign services:** Services provided in high volume with intensive demand creation (may be provided at any type of site).

**Mobile services:** VMMC services provided by a mobile team that may be delivered at an outreach site or at a non-health facility location (e.g., in tents or in a municipal building).

**Fixed site:** An established VMMC site in a health facility that provides services on a routine basis and that may also participate in campaigns.

**Outreach site:** A health facility where a team from the outside provides VMMC services—either on a regularly scheduled basis or as part of a campaign or mobile team activity.

Source: PEPFAR, 2013.^7^

Beginning in 2010, campaigns became a norm in addition to fixed site services. In campaign service delivery mode, VMMC is offered at specified sites for a specified time frame, accompanied by demand creation activities such as use of peer promoters, billboards, radio announcements, or public announcements. However, most dispensaries in which campaign services are situated are located in urban areas and within close proximity of each other. To further extend the reach of VMMC services, there was a push to scale to lower rural health facilities to reach men in more remote areas. Starting in 2014, full-time mobile outreach VMMC teams were introduced. In this highly flexible approach, mobile teams of VMMC providers, counselors, and demand creation agents with the necessary equipment (such as autoclaves and surgical tools) travel year-round to underserved areas to provide VMMC services wherever they are needed. The length of the period of service delivery in each site depends on demand at that site.

Outreach campaigns and mobile services were introduced to extend VMMC services to underserved areas.

Mobile and campaign services are time- and resource-intensive and require difficult decisions around where to place the services to reach the greatest number of potential clients. Such decisions are especially difficult when the geographic area is large, population density is low, and infrastructure is poor. To prioritize locations and populations for campaigns and mobile VMMC services in remote rural areas, MCHIP developed an approach in which project staff used GIS daily for planning mobile services, in an attempt to achieve maximum coverage.

Project staff used GIS daily to plan the location of mobile services.

### GIS and Its Application for Health

A GIS is a “computerized data management system used to capture, store, manage, retrieve, analyze, and display spatial information.”^8^ Data captured and used in a GIS can be exported and represented on digital and paper maps. GIS allows a user to represent data (also called “attributes”) referenced by their geospatial coordinates. Precise placement of administrative boundaries, roads, or terrain features can be linked to or overlaid with other data points that have been collected and enriched with latitude and longitude, or “geocoded.” Such systems provide us with ways to determine relationships between data elements that may not otherwise be obvious.

Used in many disciplines, GIS is increasingly being applied in public health. For instance, GIS has been used to track malaria risk by overlaying disease prevalence with environmental factors and types of vectors.^9^^,^^10^ Similarly, GIS has been used for predicting dengue fever^11^^,^^12^ and schistosomiasis^13^ risk. There are several, but fewer, documented uses of GIS for program implementation more generally (e.g., mapping existing service delivery locations, catchment areas, or geographic accessibility).^14^

GIS has been used in several areas of public health such as to track malaria risk and predict dengue fever.

MCHIP’s use of GIS in Tanzania evolved throughout the life of the VMMC program in 2 distinct phases, from the use of static, imperfect, and infrequently updated maps to the use of more dynamic, interactive, and iteratively updated maps.

### Phase 1: GIS and Mapping to Plan Outreach Service Locations

Our use of GIS for service delivery planning arose organically in response to implementation needs to better know the context of the areas in which outreach was being conducted and to focus our resources in areas where need or potential demand for VMMC services was highest.

**FIGURE 2:**
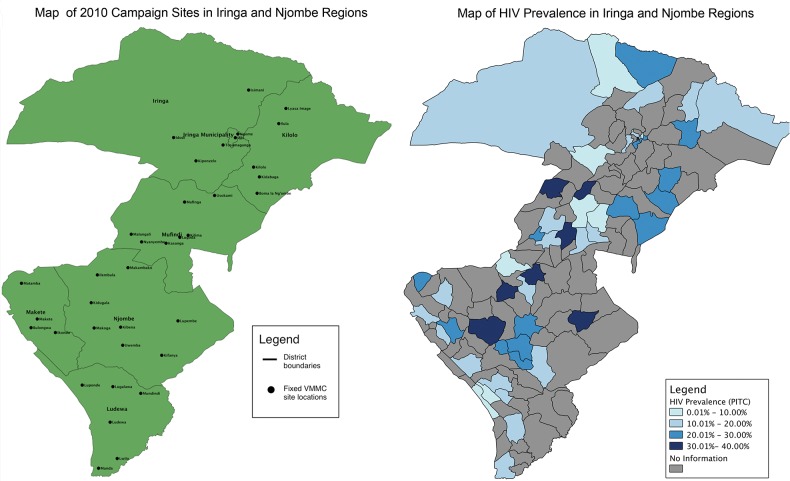
Maps Used to Plan 2012 Voluntary Medical Male Circumcision Outreach Campaign, Iringa and Njombe Regions, Tanzania

We conducted our first outreach campaigns in 2010 without the use of GIS. Later, we planned a 2012 campaign by performing a simple comparison of potential campaign site locations with HIV prevalence in Iringa and Njombe using maps of subnational administrative boundaries (Figure 2). This initial mapping used geocoded health facility data from Iringa available from previous work by the MEASURE Evaluation project,^15^ using open-source GIS software called QGIS.^16^ The mapping exercise allowed quick visual identification of areas with high HIV prevalence to target for VMMC outreach.

**FIGURE 3:**
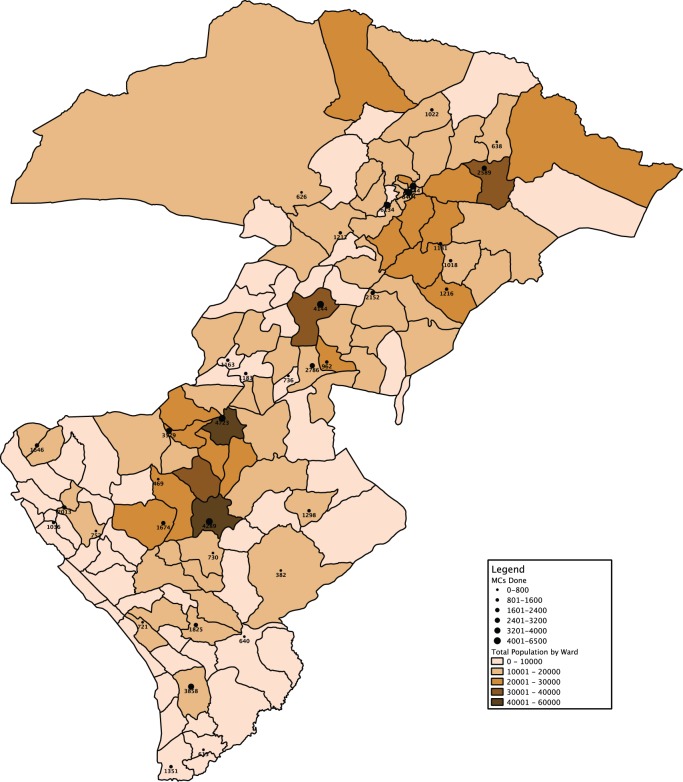
Map of Total Population Layered With Number of Voluntary Medical Male Circumcisions Performed, by Ward, Iringa and Njombe Regions, Tanzania, April 2012 Abbreviation: MC, male circumcision.

**FIGURE 4:**
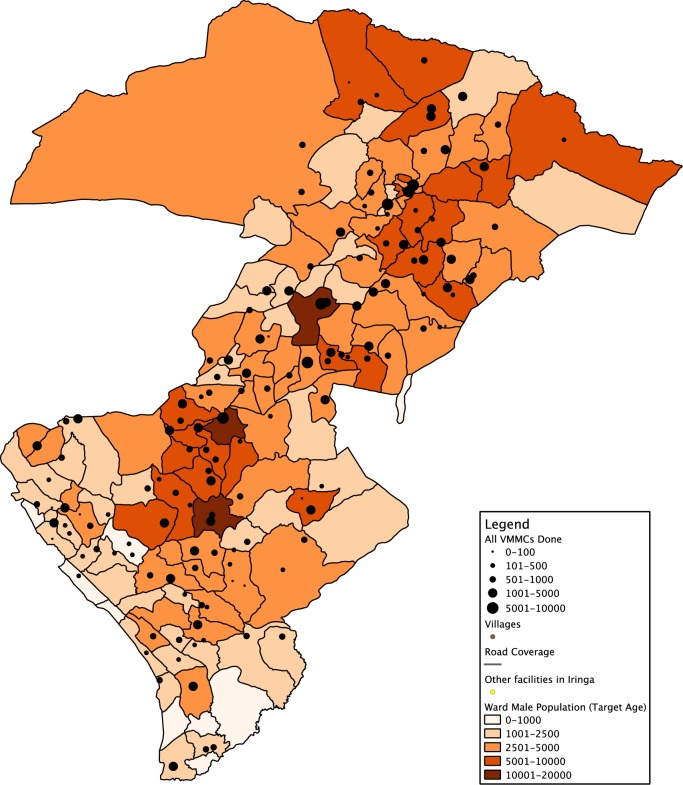
Map of Male Population Ages 10–49 Layered With Number of Voluntary Medical Male Circumcisions (VMMCs) Performed, by Ward, Iringa and Njombe Regions, Tanzania, August 2013

After we began the 2012 outreach campaigns, we combined coverage data of VMMC performed on the maps to identify locations that we had not yet reached. Maps showing projected census data reflecting total ward population were layered with points of various sizes representing facilities and the number of circumcisions performed up to that point (Figure 3). We then further refined the maps to show the number of circumcisions performed overlaid with population data specific to the target population (i.e., males aged 10–49) down to the ward level (Figure 4). The coverage estimations overlaid with population and subnational administrative boundary data were useful both for planning areas to target with VMMC outreach campaigns and for retrospectively gauging outcome measures of a particular outreach effort.

### Phase 2: Interactive Mapping With Geocoded Facility Data

With our initial success in using GIS to better understand the location of underserved populations within Iringa and Njombe, we began to envision new uses for the maps as well as areas for improvement regarding the variables fed into the maps.

For the initial GIS work, we had used coordinates collected by project staff as well as existing coordinates from publicly available data, but the coordinates from publicly collected data were frequently inaccurate. For example, maps of subnational administrative features included an undefined and incomplete data set of roads. Moreover, static subnational administrative maps overlaid with collected data did not display all of the information relevant to conducting VMMC outreach services. Program planners realized that in more remote areas where outreach was conducted, effective planning required advance knowledge of many more extenuating factors, such as accessibility via roads, availability of electricity and water, space availability at a facility, and the total catchment population of the facility.

This level of data, however, was not generally or widely available for health facilities in Iringa and Njombe regions. We thus actively sought to enrich the health facility points on the map with the necessary data on each particular facility’s infrastructure, accessibility by road, mobile network coverage, and specific populations served. To do this, we fanned out, collecting key data for mapping, including catchment population for the facility (counted from facility registers), landmarks, infrastructure, and digital photos from every health facility in Iringa and Njombe. Latitude and longitude readings were collected at every facility in the regions using inexpensive Global Positioning System (GPS) units, which were also used to track the vehicle’s path to the facility over roads that do not appear on road maps.

Development of the early maps for the project was somewhat cumbersome, requiring data entry each time a new map was to be generated and specialized skill from a monitoring and evaluation staff member to configure the map to display the desired layers. After being created, these maps were corrected or updated with new information infrequently. To address this issue, the project sought to make maps more interactive and accessible for relevant project staff.

**FIGURE 5:**
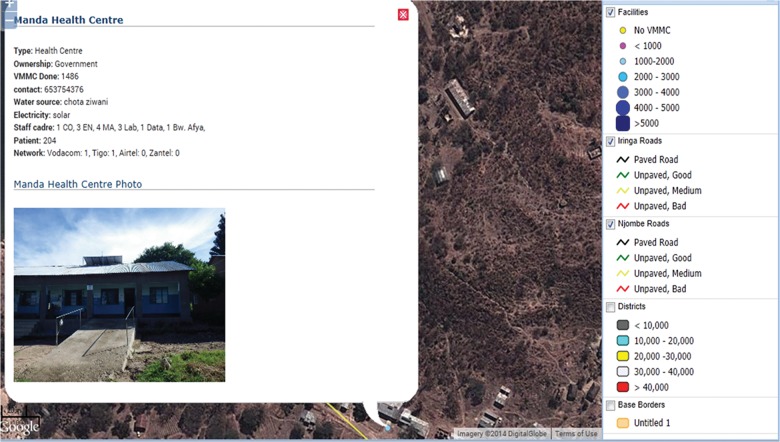
Interactive Map Displaying Information on Manda Health Centre Site, Njombe Region, Tanzania

**FIGURE 6:**
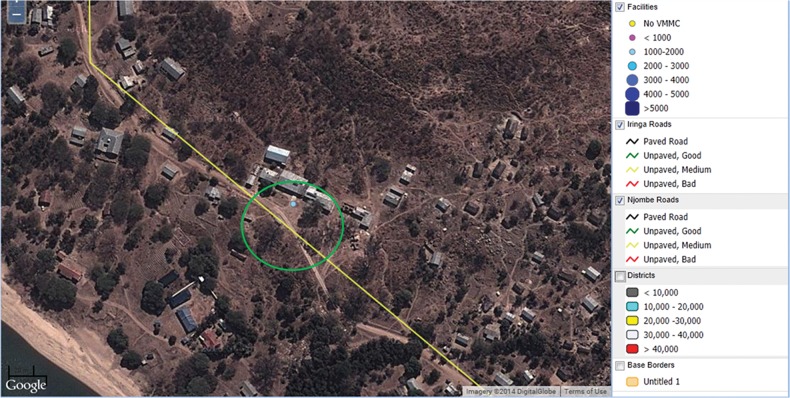
Interactive Map Displaying Satellite Imagery of Facility Layout

Using the additional geocoded data collected from facilities, the project began entering the data into a database that could be imported into Google’s free-for-use Maps Engine^17^ and, later, open-source software called OpenLayers.^18^ These applications, in contrast with the previous QGIS software, not only gave us the ability to perform the same types of overlay of facility location on population and coverage data but also allowed for interactivity with points on the map. For example, after accessing the map in an Internet browser, a user can zoom in to a particular area, click on a point, and view the specific data collected for that facility (Figure 5). Google Maps and OpenLayers also incorporate satellite imagery “basemaps”—maps depicting background reference information such as landforms, roads, and landmarks—rather than just administrative and physical boundaries (Figure 6). These satellite views further augmented the maps with visual data to inform campaign planning in terms of facility accessibility.

Using OpenLayers allowed us to link the maps directly to the project’s monitoring database, resulting in not only interactive maps but also maps that are updated as soon as new data are entered or submitted. These maps are dynamic, providing for quick turnaround between data collection and analysis of maps for program planning.

**FIGURE 7:**
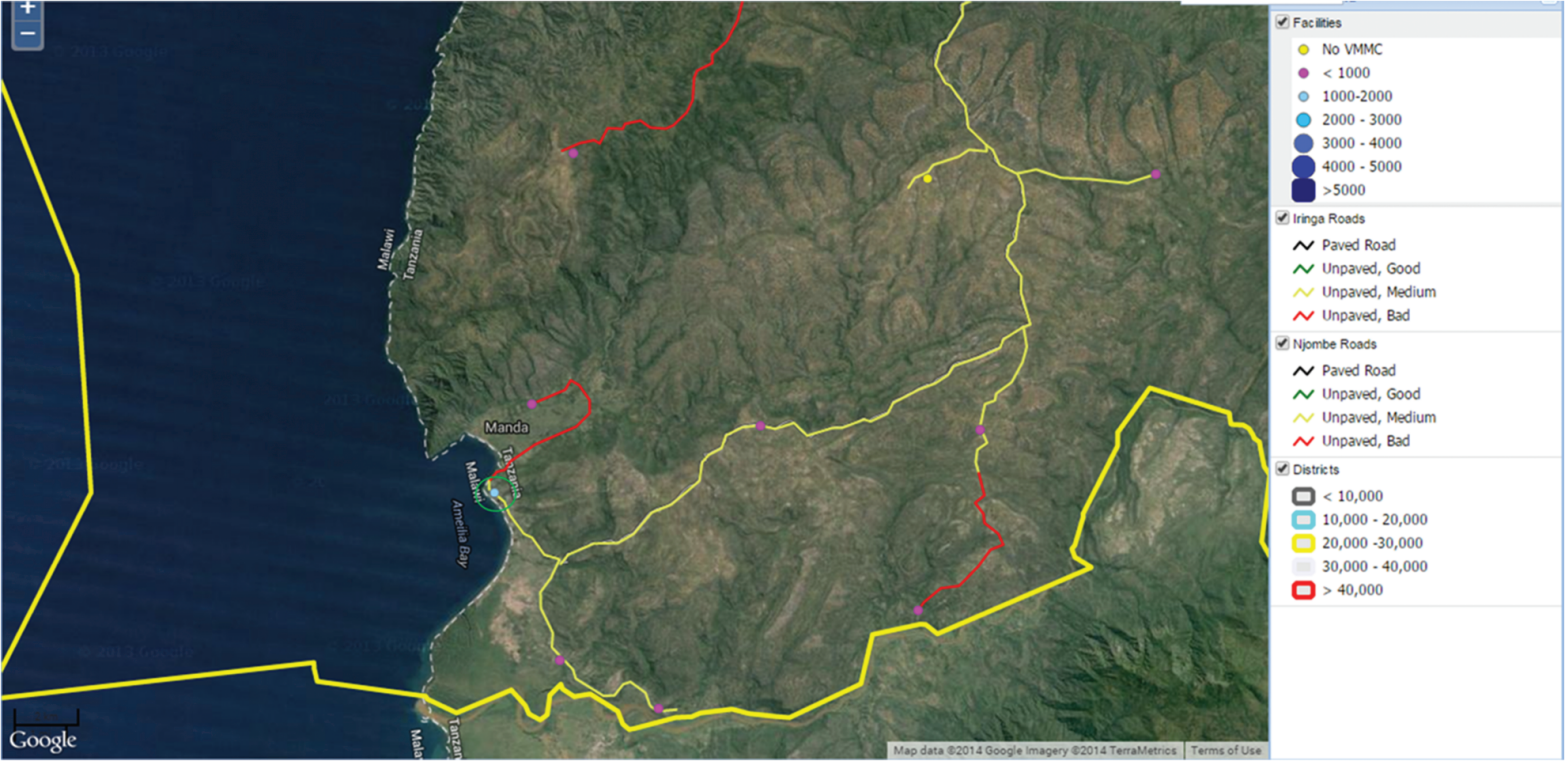
Interactive Map of Facilities Performing 0–1,000 Voluntary Medical Male Circumcisions (VMMCs)^a^ and Quality of Roads ^a^ Shown as yellow (no VMMCs) and pink (<1,000 VMMCs) circles.

With a wealth of new information, we became accustomed to using the maps on a daily basis to plan upcoming program activities. For instance, the maps from our database could be rapidly configured to show an outreach team the lower-performing facilities in a given area, for example, those that had performed between 0 and 1,000 VMMCs. Based on this information, the team could plan to focus on service provision in a particular area. Then, using the map overlay that displays road conditions, the team could make an informed decision about the area it could reach in a given time period (if at all). If roads are unpaved and in bad condition, for example, a team may decide to wait until a rainy season is over. Figure 7 illustrates one such map that displays both underserved sites and the quality of the roads that lead to them.

Retrospectively, the maps provided ways to track progress, display project monitoring data in new ways, and tell compelling stories to stakeholders about the program’s performance.

## FINDINGS

Between 2010 and 2014, the MCHIP team mapped 714 health facilities in Iringa and Njombe regions, along with relevant geocoded data—the most comprehensive mapping of health facilities in these regions to date. Using maps created from these data, MCHIP performed outreach services at 346 facility and non-facility locations during that time period.

The regional authorities of Iringa and Njombe, with the support of MCHIP, had circumcised 267,917 men by September 2014. Of these, 259,144 were aged 10-34, representing 98% of the target of 264,990. Based on the 2011–12 Tanzania HIV/AIDS and Malaria Indicator Survey (THMIS) male circumcision prevalence data and Jhpiego program data, Njombe and Iringa have gone from being among the regions with the lowest prevalence of male circumcision (29.1% at project inception^5^^,^^6^) to approaching 82% of the adult male population being circumcised, surpassing the national strategy’s regional targets.

By September 2014, the project had achieved 98% of the VMMC target in Iringa and Njombe.

**FIGURE 8:**
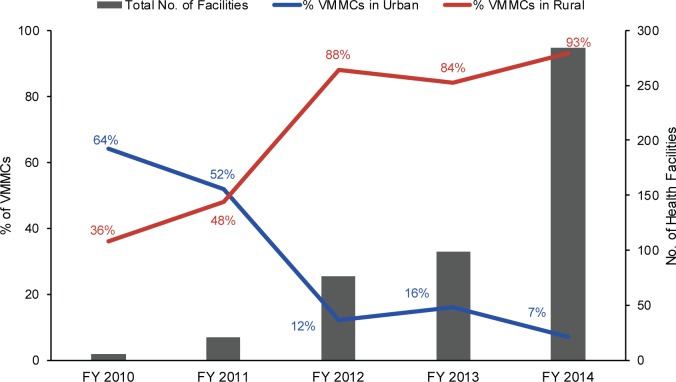
Percentage of VMMCs Performed in Urban Versus Rural Health Facilities and Total Number of Health Facilities Reached, by Fiscal Year, Iringa and Njombe Regions, Tanzania, 2010–2014

The use of GIS to collect and analyze the geographic distribution of the focus population, along with the availability of VMMC services in previously unreached areas, enabled more effective scale-up to rural lower-level health facilities by providing project staff with data that could be used to identify ideal sites for outreach. By the end of the project, nearly 4 times as many VMMCs were performed in the fiscal year than at the beginning of the project, rising from about 23,000 VMMCs in fiscal year 2010 to nearly 88,000 VMMCs in fiscal year 2014 (Table). Furthermore, the project reached substantially more men through rural dispensaries and non-health care facilities each successive year after GIS was introduced in 2012 (Figure 8).

**TABLE:** Number of VMMCs Performed in Urban Versus Rural Settings and Description of Facilities, Iringa and Njombe Regions, Tanzania, Fiscal Year 2010–2014

Year	No. of VMMCs	No. (%) of VMMCs Performed in Urban Facilities	No. (%) of VMMCs Performed in Rural Facilities	No. of Facilities	Description of Facilities
FY 2010	22,970	14,634 (64)	8,336 (36)	5	All hospitals
FY 2011	42,667	22,345 (52)	20,322 (48)	21	11 hospitals and 9 health centers
FY 2012	49,949	5,977 (12)	43,972 (88)	76	13 hospitals, 21 health centers, and 42 (lower-level) dispensaries; the 42 dispensaries contributed 46% of the achievement for the year with the hospitals and health centers contributing the remainder
FY 2013	64,407	10,069 (16)	54,338 (84)	99	10 hospitals, 13 health centers, and 76 dispensaries; dispensaries contributed 75% of the achievement for the year
FY 2014	87,924	6,125 (7)	81,799 (93)	284	12 hospitals, 24 health centers, and 248 dispensaries; dispensaries contributed 81% of the achievement for the year

Abbreviations: FY, fiscal year; VMMC, voluntary medical male circumcision.

## DISCUSSION

In Iringa and Njombe, Tanzania, use of GIS and mapping was one of several strategies to improve coverage and efficiency of VMMC scale-up. Because our use of GIS technology was part of a comprehensive planning and monitoring strategy, attribution of a particular number of circumcisions solely to our use of GIS is not possible. Without the use of GIS and digital maps, however, our provision of VMMC outreach services would have been far less focused on areas with specific need and potentially more costly, for example, if outreach teams were deployed to sites where unmet need was low or infrastructure was unavailable.

### Using GIS to Reach Clients in the “Last Kilometer”

The global health community frequently invokes the concept of the “last mile” or, in the case of Tanzania, the “last kilometer” to refer to achieving coverage for those clients who are the most difficult to reach. In terms of our VMMC outreach campaigns, the necessity to reach clients in the “last kilometer” was literal. For VMMC to reach highest public health impact for HIV prevention, rapid scale-up of services is necessary even in the most remote and difficult-to-access areas. Without the use of GIS, programmatic decisions regarding where to locate outreach services would have been broadly conceptualized: the program would have simply held campaigns in areas of Iringa and Njombe where male circumcision rates were low. By using GIS, we were able to fine-tune our outreach effectiveness, making decisions considering age of population versus circumcision rates, catchment areas of facilities where our teams had already provided outreach, accessibility of facilities where outreach was performed, and which model of service would best suit the level of infrastructure (e.g., using mobile units in areas with potential demand but no health facilities).

Using GIS allowed us to fine-tune our outreach campaigns.

### Using GIS Data for Decision Making

Mapping has long been recognized as an important public health tool, hearkening back to the classic anecdote about John Snow, the Broad Street pump, and the cholera outbreak.^19^ Frequently, however, use of maps for public health stops after the simple plotting of points. Latitude and longitude remain simply coordinates without other geocoded data to which we can link those points.

By dispatching teams to collect relevant data about health facilities’ infrastructure, accessibility, and catchment areas, we were able to create dynamic, interactive maps that informed our program planning and served as a monitoring tool for our outreach efforts. Once a facility is geocoded and present in the database, it is possible to attach any number of variables to that record in the database. This approach has applicability to a wide variety of public health services, especially those which require outreach services.

### Replicating and Expanding the Approach

After the success with GIS for VMMC scale-up in Iringa and Njombe, the project is using the same approach to scale-up VMMC in Tabora region of Tanzania. GIS work in Tabora will leverage the same methods for data collection and mapping established for Iringa and Njombe; initial steps will be collecting facility data and synchronizing it with our existing database. Since the mapping features of OpenLayers are interactive and represent the entire world, not just the areas where we have worked, all the data, from Iringa and Njombe as well as Tabora, will be available to program staff and filterable by data elements of any uploaded data point, allowing cross-regional analysis.

Many potential applications for the use of GIS beyond VMMC exist, particularly for programs that require outreach and are attempting coverage of a particular age strata or population type, such as planning and mapping outreach efforts for vaccination campaigns, where the children of eligible age can be quantified using local government records. The type of mapping facilitated by GIS can also be used to track coverage and service delivery of HIV care and treatment services, as well as many other service delivery areas. Our project’s particular maps are available to any organization implementing health activities in Iringa and Njombe regions.

## LESSONS LEARNED

### GIS and Mapping Software Selection

The approach to using GIS detailed in this article arose organically, with no particular approach for selecting software. This is not necessarily a best practice, and the project ultimately switched software several times to better meet project needs. In future implementations of GIS to improve public health program implementation, requirements for software and mapping functionality should be laid out in advance of selecting software.

Moreover, while we tout the free-for-use nature of much of the software we used as a benefit, we also note that such software can come with caveats. For instance, the Google software service agreement gives Google the right to access data within limited purposes of “operating, promoting, and improving our services, and to develop new ones”; however, the user retains intellectual property rights over the data.^20^ For the purposes of our project, in which only facility-level/aggregate data were collected and uploaded, the project felt that this service agreement was acceptable. However, such privacy and security concerns are an important factor in the decision to use any type of software. Security of data in cloud-based systems particularly is an issue, especially if a project wishes to collect and/or map patient-level data.

### Cost Implications of GIS Implementation

While we described many ways in which we believe GIS allowed the project to be more efficient in allocating resources, planning for start-up costs, or merely the anticipated high cost of GIS, could be perceived as barriers to use by program managers. Any project using GIS requires initial setup of a database, data collection, deployment of GIS software, and initial site mapping (if no geocoded site data already exist)—all of which have costs associated with them and could be perceived as barriers. We minimized many of the costs associated with these activities by incorporating them into our normal project monitoring and evaluation activities and level of effort allocated for project staff. However, the time necessary for start-up data collection and mapping can be substantial; sharing existing map data among projects at the country level would be advantageous to reduce costs in this area.

## CONCLUSION

GIS was a useful tool to prioritize delivery of VMMC services in specific areas and to particular population groups, providing maximum effectiveness for scale-up in 2 regions of Tanzania, and it ensured that supply of services matched demand. After incorporation of GIS into the VMMC program for strategic planning, the MOHSW with MCHIP support was able to double regional targets for VMMC, improving efficiencies that have the potential for both epidemiologic and cost benefits for the program. Use of GIS can be an effective means to make strategic decisions about service delivery and coverage, especially in the context of mobile and outreach services, for other VMMC programs as well as for other areas of health service delivery. In the future, ministries of health and donors may want to consider funding mapping initiatives that support numerous interventions, spreading any initial start-up or data collection costs among implementing partners rather than placing a burden on each intervention individually. Although this approach will require coordination among stakeholders, the benefits of geospatial coordination may be great for country programs.

Use of GIS can be an effective means to make strategic decisions about service delivery and coverage.

Health information system platforms that include GIS and digital mapping capabilities as part of their core functionality, such as the District Health Information System 2 (DHIS2), are becoming increasingly widespread in low- and middle-income countries. Correspondingly, geocoded data and spatial analysis of health service delivery data will be increasingly important. Incorporating GIS into programmatic efforts paves the way for smarter, more responsive, and more cost-efficient public health planning, increasing the likelihood of appropriate and effective use of public health resources.

## Notes

**Peer Reviewed**
